# A Novel High-Resolution and Sensitivity-Enhanced Three-Dimensional Solid-State NMR Experiment Under Ultrafast Magic Angle Spinning Conditions

**DOI:** 10.1038/srep11810

**Published:** 2015-07-03

**Authors:** Rongchun Zhang, Manoj Kumar Pandey, Yusuke Nishiyama, Ayyalusamy Ramamoorthy

**Affiliations:** 1 Biophysics and Department of Chemistry, University of Michigan, Ann Arbor, MI 48109-1055, USA; 2 RIKEN CLST-JEOL collaboration center, RIKEN, Yokohama, Kanagawa 230-0045, Japan; 3 JEOL RESONANCE Inc., Musashino, Akishima, Tokyo 196-8558, Japan

## Abstract

Although magic angle spinning (MAS) solid-state NMR is a powerful technique to obtain atomic-resolution insights into the structure and dynamics of a variety of chemical and biological solids, poor sensitivity has severely limited its applications. In this study, we demonstrate an approach that suitably combines proton-detection, ultrafast-MAS and multiple frequency dimensions to overcome this limitation. With the utilization of proton-proton dipolar recoupling and double quantum (DQ) coherence excitation/reconversion radio-frequency pulses, very high-resolution proton-based 3D NMR spectra that correlate single-quantum (SQ), DQ and SQ coherences of biological solids have been obtained successfully for the first time. The proposed technique requires a very small amount of sample and does not need multiple radio-frequency (RF) channels. It also reveals information about the proximity between a spin and a certain other dipolar-coupled pair of spins in addition to regular SQ/DQ and SQ/SQ correlations. Although ^1^H spectral resolution is still limited for densely proton-coupled systems, the 3D technique is valuable to study dilute proton systems, such as zeolites, small molecules, or deuterated samples. We also believe that this new methodology will aid in the design of a plethora of multidimensional NMR techniques and enable high-throughput investigation of an exciting class of solids at atomic-level resolution.

There is a significant need for techniques that can provide atomic-level structural and dynamics information from numerous non-soluble and non-crystallizable systems such as bone^1^,^2^, amyloid fibrils^3^,^4^,^5^,^6^,^7^,^8^, membrane proteins^9^,^10^,^11^,^12^,^13^,^14^ and nanomaterials^15^,^16^. The most commonly used high-resolution techniques like solution NMR and X-ray crystallography cannot be used to study most of these systems. Although solid-state NMR spectroscopy has been demonstrated as a main technique for atomic-level characterization of such systems, very poor sensitivity has limited its applications^17^,^18^,^19^. While magic angle spinning (MAS) techniques have enhanced spectral resolution and sensitivity, direct detection of the most sensitive nucleus, proton, due to large dipolar couplings among protons, was not possible^20^,^21^. Luckily, recent advent of ultrafast MAS probes enabled the development of proton-detected solid-state NMR techniques^22^,^23^,^24^,^25^,^26^,^27^,^28^,^29^,^30^,^31^ as well as the discovery and demonstration of new NMR phenomena under ultrafast MAS^32^,^33^,^34^,^35^,^36^,^37^. In this study, we demonstrate the use of proton-detection under ultrafast MAS conditions to develop multidimensional experiments that can further enhance the spectral resolution. Specifically, a three-dimensional technique that correlates single-quantum (SQ) and double-quantum (DQ) coherences of protons is successfully demonstrated for studies on biological solids. The 3D spectrum also provides the SQ/SQ correlation of proton chemical shifts via proton-proton dipolar couplings recoupled by the fp-RFDR (finite-pulse radio frequency driven dipolar recoupling) pulse sequence^38^,^39^. Furthermore, it also correlates the DQ coherence with indirectly (*t*_1_ dimension) and directly (*t*_3_ dimension) detected SQ coherences, both of which provide complementary and abundant information about proton proximities in addition to the 2D ^1^H/^1^H SQ/SQ correlation spectrum. Preparation, evolution and detection of protons not only enhance the overall sensitivity of the experiment, but also enable the implementation of this technique on most NMR spectrometers without the need for multiple RF channels. Powder samples of U-^13^C-^15^N-L-alanine and N-acetyl-L-^15^N-valyl-L-^15^N-leucine (NAVL) are used as model systems to demonstrate the proposed 3D pulse sequence in this study. Experimental results obtained at two different spinning speeds (60 and 90 kHz) are reported, and advantages and limitations of the 3D technique are also discussed.

## Results and Discussion

### 3D SQ/DQ/SQ NMR pulse sequence

**Figure 1:**
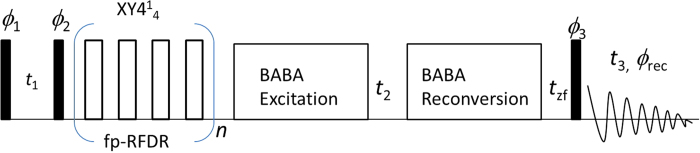
3D SQ/DQ/SQ pulse sequence. Radio-frequency pulse sequence for proton-based 3D solid-state NMR experiment that correlates single quantum, double quantum and single quantum coherences under ultrafast MAS conditions. While ultrafast MAS suppressed most line broadening interactions, including proton-proton dipolar couplings, fp-RFDR with an 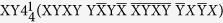 phase cycling is used to recouple proton-proton dipolar couplings. Broadband BABA^41^ or BABA-XY16^40^ sequence was employed for the DQ excitation and reconversion depending on the sample investigated. A short z-filter delay (*t*_zf_) is inserted right before the 90^o^ read pulse for removing any residual transverse magnetization. For experiments on L-alanine, the following phases were used: *ϕ*_1_ = 02; *ϕ*_2_ = 00220022; *ϕ*_3_ = 0000, *ϕ*_rec_ = 0202. Experiments on NAVL employed the following phase cyclings: *ϕ*_1_ = 0; *ϕ*_2_ = 4(0)4(180); *ϕ*_3_ = 8(0), 8(120), 8(240); *ϕ*_rec_ = 2(0, 180), 2(180,0), 2(120, 300), 2(300, 120), 2(240, 60), 2(60, 240).

The 3D radio-frequency pulse sequence proposed in this study is shown in Fig. 1. Proton magnetization is prepared by a 90° RF pulse first, and then the transverse proton magnetization is allowed to evolve under isotropic chemical shifts of protons during an incrementable *t*_*1*_ period. After the *t*_1_ period, the longitudinal magnetization generated by a 90° RF pulse is allowed to exchange under ^1^H-^1^H dipolar couplings recoupled by the fp-RFDR pulse sequence during the mixing period. The fp-RFDR pulse sequence consists of rotor-synchronized 180° pulses (*i.e., τ*-180°−*τ* = one MAS rotor period) that are phase cycled according to 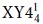, which was recently demonstrated to be tolerant to pulse imperfections such as chemical shift offset and RF field inhomogeneity^33^,^34^. Following the fp-RFDR mixing period, SQ coherence is converted to DQ coherence by the back-to-back (BABA)^40^,^41^ pulse sequence, and the DQ coherences of protons are allowed to evolve during the RF-free incrementable *t*_2_ period. After expressing the DQ frequency, the DQ coherences are converted back to SQ coherences by the BABA pulse sequence. Then, a 90° read pulse is applied to acquire the transverse magnetization of protons during the *t*_3_ period. Generally a short *z*-filter delay is inserted right before the 90° read pulse to remove the residue magnetization on the transverse plane. The pulse sequence is repeated after a relaxation/recycle delay for signal averaging to increase the signal-to-noise ratio as well as to satisfy the phase cycling (given in Fig. 1) used to obtain DQ coherence signal and suppress any artifacts in the spectrum.

### 3D SQ/DQ/SQ spectrum of L-alanine at 60 kHz MAS

**Figure 2:**
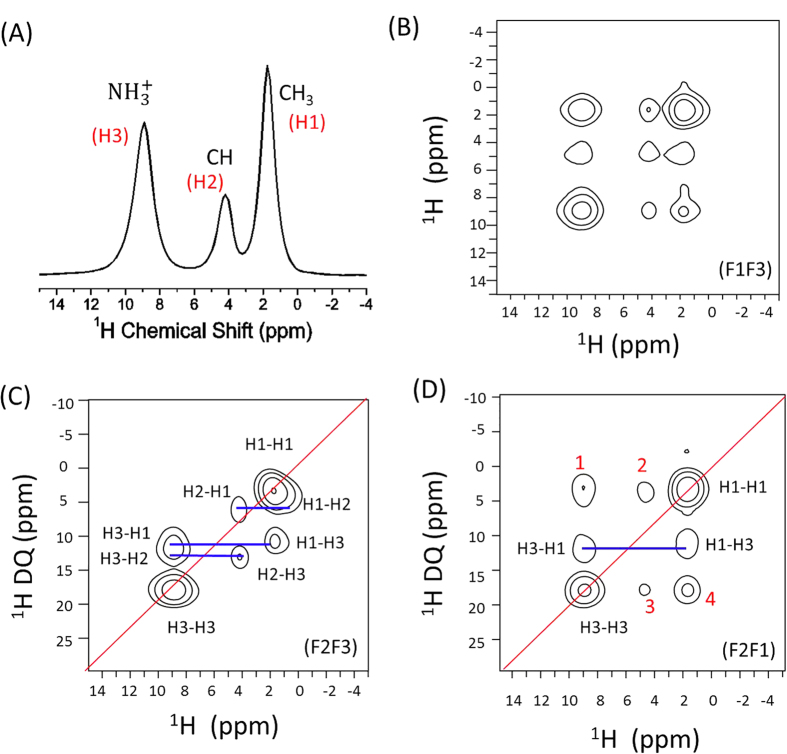
High-resolution ^1^H ultrafast MAS spectra of L-alanine. Single pulse ^1^H NMR spectrum (**A**) and 2D spectral slices extracted from the 3D SQ/DQ/SQ spectrum (**B**,**C**,**D**) of L-alanine powder sample under 60 kHz MAS. A 1 ms fp-RFDR mixing time was used, and broadband BABA sequence was utilized for the DQ excitation/reconversion with a time duration of 66.7 μs.

Experiments were first performed on a powder sample of U-^13^C,^15^N-L-alanine at 60 kHz MAS to demonstrate the feasibility of the above-mentioned 3D pulse sequence and the results are given in Fig. 2. The proton ultrafast-MAS spectrum of L-alanine shown in Fig. 2A displays well resolved peaks that are assigned to three chemically different protons associated with CH_3_, CH and 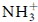 groups. Three different 2D spectral slices extracted from the 3D spectrum of alanine are shown in Fig. 2(B–D). As indicated in Fig. 2, correlations of frequencies expressed in *t*_1_ and *t*_3_, *t*_2_ and *t*_3_, and *t*_2_ and *t*_1_ periods of the 3D experiment are shown in 2D SQ1/SQ2, 2D DQ/SQ2, and 2D DQ/SQ1 in Fig. 2B–D, respectively; SQ1 is the single quantum coherence expressed during *t*_1_ whereas SQ2 during *t*_3_. The 2D SQ1/SQ2 spectrum shown in Fig. 2B consists of diagonal and cross peaks, and the observed cross peak pattern indicates that a 1 ms fp-RFDR mixing is sufficient to accomplish a total correlation of resonances. The 2D DQ/SQ2 spectrum (Fig. 2C) shows the proximities of different protons in alanine at a high resolution in the indirect DQ dimension, as the spectral span of the DQ dimension is twice that of the SQ dimension. A spin pair close in proximity generates a DQ signal within a proper BABA excitation time, exhibiting a pair of peaks having the same chemical shift value – that is the sum of the isotropic chemical shifts of the two different spins in the SQ dimension - in the DQ dimension. The 2D DQ/SQ1 spectrum shown in Fig. 2D provides the same information as the 2D DQ/SQ2, but it also reveals additional information about the proton proximity. In addition to the peaks displayed in the 2D DQ/SQ2 spectrum, the 2D DQ/SQ1 spectrum also consists of peaks (1,2,3,4) in Fig. 2D, which are not symmetric with respect to the diagonal line. Specifically, a cross peak in the 2D DQ/SQ1 indicates the proximity between a pair of protons that contribute to the signal observed in the DQ dimension and the proton corresponding to the peak in the SQ dimension, as the DQ coherence is generated right after the fp-RFDR based dipolar-coupling driven magnetization exchange. For example, if proton A involved in magnetization exchange through fp-RFDR with proton B, while protons B and C are close enough to induce a DQ coherence signal, then a cross peak appears with a frequency of proton A chemical shift in the SQ1 dimension and with a frequency of the sum of the chemical shifts of protons B and C in the DQ dimension. Therefore, the proximities between 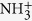 and (CH_3_-CH_3_ pair), CH and (CH_3_-CH_3_ pair), CH and (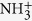-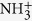
pair), and CH_3_ and (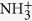-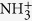
pair) protons are revealed by the peaks 1, 2, 3 and 4, respectively, in Fig. 2D.

It is worth mentioning that not all the DQ peaks observed in the 2D DQ/SQ2 (F2/F3) spectrum appear in the DQ/SQ1 (F2/F1) spectrum. For example, H2-H1 and H2-H3 DQ peaks that appear in Fig. 2C are missing in Fig. 2D. This is mainly because the 2D spectra given in Fig. 2 were obtained through skyline projection along the third dimension. In the 2D DQ/SQ1 (F2/F1) spectrum, proton signals largely decayed after the chemical shift evolution in the *t*_1_ period and the fp-RFDR mixing period even before the excitation of DQ coherences. On the other hand, for the 2D DQ/SQ2 (F2/F3) spectrum, all the DQ coherences in the *t*_2_ period are transformed to an observable SQ2 coherence. However, the missing information could be found back in the 2D DQ/SQ1 spectra sliced at different proton chemical shifts along the F3 dimension as shown in Fig. S1, where only the relevant peaks appear in the sliced 2D spectra.

### 3D SQ/DQ/SQ spectrum of NAVL at 90 kHz MAS

**Figure 3:**
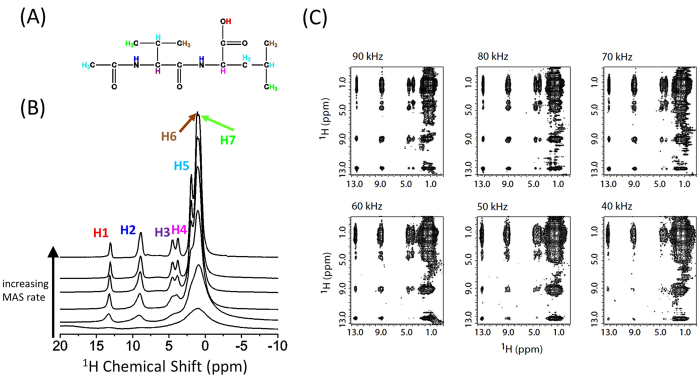
Increasing spinning speed dramatically enhances the proton spectral resolution. (**A**) Chemical structure; (**B**) 1D ^1^H NMR spectra of NAVL obtained at MAS speeds of 10, 20, 40, 60, 80, and 90 kHz from bottom to top; (**C**) 2D ^1^H/^1^H fp-RFDR^33^,^34^ correlation spectra obtained at the indicated spinning speeds. As seen from the spectra, enhancement of spectral resolution is achieved by increasing the MAS speed. The fp-RFDR mixing time was 2.84 ms. 64 *t*_1_ increments were used.

**Figure 4:**
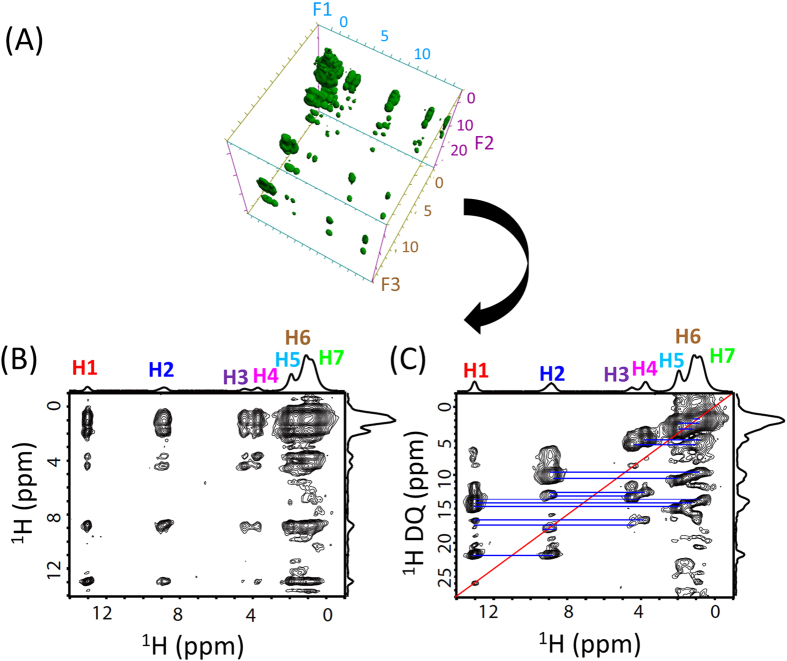
High-resolution 3D and 2D ultrafast MAS spectra of NAVL. (**A**) 3D SQ/DQ/SQ spectrum of NAVL powder sample obtained at 90 kHz MAS. (**B**) 2D F1/F3 and (**C**) F2/F3 spectra extracted from the 3D spectrum. A 2.84 ms fp-RFDR mixing and the BABA-xy16^40^ sequence with 88.9 μs excitation/reconversion time were used. The DQ peaks are indicated with the blue lines.

**Figure 5:**
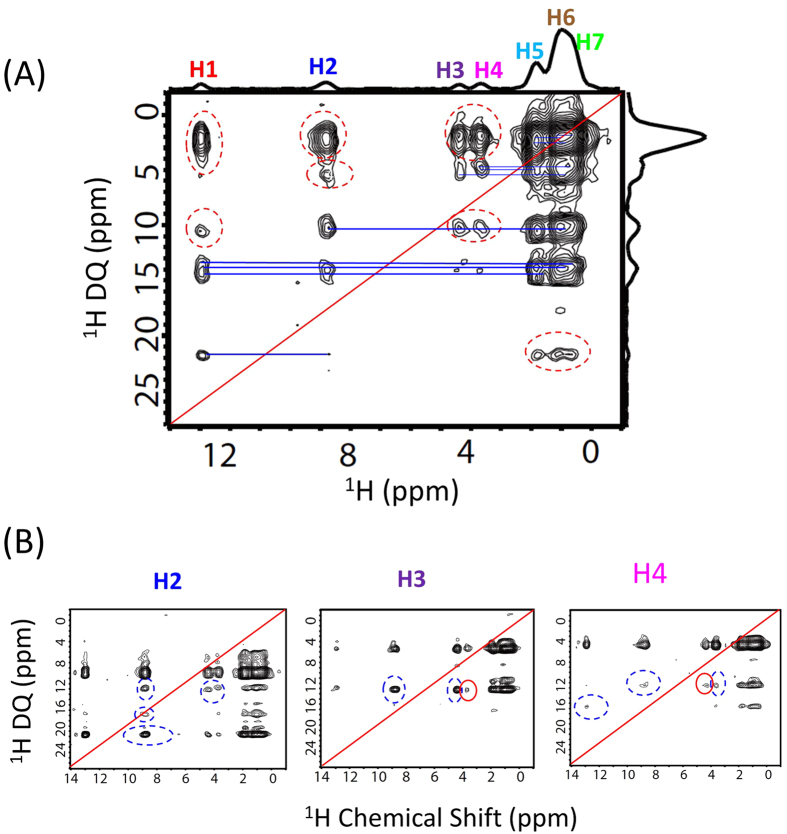
High-resolution 2D F2/F1 ultrafast MAS spectra extracted from the 3D SQ/DQ/SQ spectrum of NAVL. (**A**) F2/F1 skyline projection of the ultrafast MAS 3D spectrum, where the blue lines indicate the normal DQ peaks, while the red dashed circles indicate the peaks absent in the general DQ/SQ (F2/F3) spectrum (see Fig. 4B). (**B**) 2D F2/F1 ultrafast MAS spectra sliced at different chemical shift frequency along the F3 dimension as indicated, where the new cross peaks indicated by the blue dashed circles and red solid circles are absent in Fig. 5A. Other 2D F2/F1 spectra sliced at other chemical shifts are shown in Figure S2.

To further demonstrate the feasibility of the 3D experiment presented in this study, we carried out measurement on a powder sample of N-acetyl-L-^15^N-valyl-L-^15^N leucine (NAVL) under 90 kHz MAS. The chemical structure and a series of 1D proton chemical shift spectra of NAVL obtained at different spinning speeds are given in Fig. 3A,B. The 1D spectra clearly demonstrate the increase in spectral resolution with the increasing spinning speed of the sample. In particular, the quite overlapped H5, H6 and H7 (as indicated in Fig. 3B) are well resolved for spinning rates beyond 60 kHz. The spectra obtained at 90 kHz spinning speed show very well resolved narrow peaks that are assigned to chemically different protons in the molecule. A clear demonstration of the spectral resolution enhancement can be also seen in the 2D ^1^H/^1^H correlation spectra recorded at MAS rates in the range of 40 to 90 kHz, as shown in Fig. 3C. In these measurements, a total correlation among protons was achieved within 2.84 ms mixing time. Based on these results, we performed the 3D SQ1/DQ/SQ2 experiment and the resultant 3D spectrum along with the 2D SQ1/SQ2 (F1/F3) and DQ/SQ2 (F2/F3) correlation spectra projected from the 3D spectrum are shown in Fig. 4. The 2D SQ2/SQ1 (F3/F1) spectral slice in Fig. 4B shows that a fp-RFDR mixing time of 2.84 ms is sufficient to accomplish a total correlation of proton resonances in NAVL, which in principle is similar to a normal 2D ^1^H/^1^H correlation spectrum as shown in Fig. 3C except for the intensities of cross peaks. The 2D DQ/SQ2 (F2/F3) spectrum extracted from the 3D spectrum provides the proximity information at a higher resolution compared to that rendered by the 2D SQ1/SQ2 correlation spectrum due to the double spectral span in the DQ dimension. In particular, additional information could be extracted from the 2D DQ/SQ1 spectra as shown in Fig. 5. As seen from Fig. 5A, new cross peaks are observed from a 2D DQ/SQ1 spectrum directly projected from the 3D spectrum (Fig. 4A) in comparison to the 2D DQ/SQ2 (F2/F3) spectrum shown in Fig. 4C. Most importantly, these new cross peaks indicate the proximity between a spin and a certain other pair of spins that are close enough to induce a DQ signal via the recoupled ^1^H-^1^H dipolar couplings. It is worthwhile to mention here that few cross peaks (as indicated by the dashed red circles) in the DQ/SQ2 (F2/F1) spectrum are not observed in the projected 2D DQ/SQ2 (F2/F3) spectrum. Nevertheless, these missing cross peaks can be observed in a 2D DQ/SQ1 spectral slice obtained from the 3D spectrum at an isotropic chemical shift frequency in the F3 dimension, as indicated by dashed blue circles and red solid circles in Fig. 5B. In fact, the 2D spectral slices shown in Fig. 5B did provide a better resolved 2D DQ/SQ1 spectrum, as they only provide local information related to the protons at the specific chemical shift frequency at which the 2D slice is extracted. For example, if the 2D slice is taken at the isotropic chemical shift value of H1, then the cross peaks correlating with H1 will only be observed. Subsequently, we can see the DQ cross peaks between H1 and other protons in the vicinity (H2, H3, H4, H5, H6, and H7) which are also observed in the 2D DQ/SQ2 (F2/F3) spectrum, as shown in Figures S2A and 4C. Besides, as explained above, there are also some cross peaks, which do not have their symmetric counterpart parts below or above the diagonal line. As explained above, these peaks actually indicate the proximity between a spin (indicated by the chemical shift frequency along the F1 dimension) and a pair of spins (indicated by the chemical shift frequency along the F2 dimension).

The proposed 3D experiment could provide more accurate information about proton proximities especially when proton peaks are overlapped, which is very often the case even under ultrafast MAS conditions. For example, let us assume a hypothetical molecule that exhibit three well resolved peaks (A, B and C) in a 1D proton NMR spectrum under ultrafast MAS conditions; and suppose the correlation peaks of A–B and A–C are observed, while the B–C correlation peak is absent, in the regular 2D DQ/SQ spectrum. Such spectral information arises from the following arrangement of protons: B-A-C. However, if the peak A is an overlap of resonances from two different types of protons (A1 and A2), then there could be two possible structures: (a) B-A1 and A2-C and (b) B-A1-A2-C or A1-B-A2-C. A regular 2D DQ/SQ spectrum cannot differentiate these two possible structures, but they could be identified from a 3D spectrum. By taking the 2D DQ/SQ1 spectral slice at the chemical shift frequency of B, we could only observe a correlation peak at (δ_B_ + δ_A1_, δ_B_) for the structure (a), as observed from the regular 2D DQ/SQ spectrum. However, for the structure (b), an additional peak at (δ_B_ + δ_A2_, δ_C_) can be observed due to the magnetization exchange between protons A2 and C nuclei through fp-RFDR. This is indeed observed for NAVL sample in this study. In the 2D DQ/SQ2 (F2/F3) spectrum obtained from the skyline projection of the 3D spectrum of NAVL, as shown in Fig. 4C, correlations between H2 (amide proton) and H3 as well as H2 and H4 are observed, while the H3-H4 correlation peak is not observed. Therefore, the above-mentioned two possible structures (i.e., a and b) could not be distinguished simply from a regular (or the projected) 2D DQ/SQ spectrum. However, the 2D DQ/SQ1 spectral slices taken at the chemical shift frequencies of H3 and H4 nuclei (shown in Fig. 5B) display additional peaks, at (δ_H2b_ + δ_H3_, δ_H4_) and (δ_H2b_ + δ_H4_, δ_H3_) respectively, as indicated by the red solid circles. These additional correlation peaks arise from the structure (b). Indeed, the protons in NAVL arrange as H2a-H3-H2b-H4 (i.e., structure (b)). The peak observed at (δ_H2b_ + δ_H4_, δ_H3_) has a stronger intensity than that at (δ_H2b_ + δ_H3_, δ_H4_) because the DQ intensity induced by H2b and H3 is weaker than that induced by H2b and H4 due to the longer distance between H2b and H3 than that between H2b and H4. Therefore, the 2D spectral slices extracted from the 3D spectrum can be used for spectral editing, which can be used to enhance spectral resolution and to assign resonances.

It is worth noting here that ^1^H spectral resolution is the key to the success of the 3D experiment. As well demonstrated in Fig. 3, the spectral resolution increases with the spinning speed of the sample. For example, peaks for H3 and H4 and associated cross peaks in 2D spectra are well resolved for higher spinning speeds (>60 kHz). Although ultrafast MAS renders excellent line narrowing and thus enhances spectral resolution and sensitivity,^42^,^43^,^44^,^45^ it is still difficult to fully resolve all ^1^H spectral lines for a system with a dense proton dipolar coupled network. CRAMPS (Combined Rotation And Multiple-Pulse Sequence) approach^46^,^47^ could be used to further suppress ^1^H-^1^H dipolar couplings, though at the expense of signal-to-noise ratio. However, for a system with dilute protons, such as zeolites, metal-organic framework (MOF), or deuterated molecules, a high proton spectral resolution could still be easily achieved. In particular, deuteration is necessary for high-resolution structural studies on proteins or other biomolecules as demonstrated in the literature^48^,^49^,^50^. We also would like to point out other practical difficulties associated with strong water ^1^H peak, distribution of ^1^H-^1^H dipolar coupling values, and the distribution of spin-lattice (*T*_*1*_) relaxation values for protons. Fortunately, water suppression could be easily achieved by solvent suppression schemes^45^,^51^ in the very beginning of the 3D pulse sequence. On the other hand, the distributions of ^1^H-^1^H dipolar coupling and *T*_*1*_ values could be a problem in the selection of a suitable fp-RFDR mixing time for the 3D experiment. Indeed, in order to achieve total ^1^H/^1^H correlations with good signal-to-noise ratio, the choice of fp-RFDR mixing time is quite important. As reported in our previous study^34^, the rate of proton magnetization transfer under fp-RFDR increases with the ^1^H-^1^H dipolar coupling value or the duty factor (the ratio of π pulse length to the rotor period) of the fp-RFDR sequence. For most rigid solid systems, a few milliseconds of mixing time is sufficient to achieve total chemical shift correlation of protons; whereas, a longer mixing time (for example, tens of milliseconds) is essential for semi-solids^52^. In any case, since a 2D ^1^H/^1^H SQ/SQ experiment can be performed very quickly, an optimization of the fp-RFDR mixing time for maximum sensitivity and optimum spectral correlation is highly recommended for chosen spinning speed and RF power.

## Conclusions

In summary, we have demonstrated the advantages of using ultrafast MAS for enhancing the proton spectral resolution and sensitivity. In particular, with the utilization of fp-RFDR for ^1^H-^1^H dipolar recoupling and BABA for DQ coherence excitation/reconversion, a high-resolution and sensitivity-enhanced proton-based 3D sequence is proposed, which correlates DQ and SQ coherences and provides abundant information about the proton proximities in solids. Our results show that, besides the general 2D ^1^H/^1^H SQ1/SQ2 (F1/F3) and DQ/SQ2 (F2/F3) correlation spectra, the third 2D DQ/SQ1 (F2/F1) spectrum extracted from the 3D spectrum reveal additional information that can potentially be useful to measure proton-proton distances. The cross peaks observed in the 2D DQ/SQ1 (F2/F1) spectrum not only contain the general DQ/SQ correlation information, but also provide additional information about the proximity between a spin and a certain other dipolar coupled pair of spins. The spectral resolution rendered by the 2D spectral slices - extracted from the 3D spectrum - can be used to overcome the difficulties in assigning peaks observed in a 2D ^1^H/^1^H RFDR-based chemical shift correlation spectrum. Thus, this approach could be useful to determine distances between protons using high-throughput RFDR based proton-detected experiments under ultrafast MAS conditions. Therefore, we believe that this new proton-based 3D ultrafast MAS solid-state NMR technique will aid in the design of a plethora of multidimensional NMR pulse sequences and enable a high-throughput investigation of dynamics based function of an exciting class of solids at atomic-level resolution.

## Methods and Materials

### Samples

Uniformly ^13^C, ^15^N-L-alanine was purchased from Isotec (Champaign, IL) and directly used as received without any purification. A powder sample of N-acetyl-^15^N-L-valyl-^15^N-L-leucine (NAVL) was prepared as explained elsewhere^53^.

### *NMR* Experiments

Experiments on L-alanine were performed on an Agilent VNMRS 600 MHz solid-state NMR spectrometer using a triple-resonance 1.2 mm MAS probe (Varian/Agilent) operating at 599.8 MHz for ^1^H. 90^o^ pulse length was 1.5 μs. The fp-RFDR pulse sequence with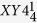 phase cycling^33^,^34^ was used for recoupling ^1^H-^1^H dipolar couplings in the mixing time. A 3 μs z-filter delay before the final read pulse was used. A broadband BABA^41^ sequence for a duration of 66.7 μs was employed for the DQ signal excitation and reconversion. A recycle delay of 3 s was used. All NMR experiments on NAVL were performed on a 600 MHz ECZ600R solid-state NMR spectrometer equipped with a 0.75 mm double-resonance ultrafast MAS probe (JEOL RESONANCE Inc.). A 0.6 μs 90^o^ pulse length was used. In the 3D SQ/DQ/SQ experiment, a finite-pulse RFDR pulse sequence with 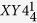 phase cycling scheme was used to recouple ^1^H-^1^H dipolar couplings for a mixing time of 2.84 ms. BABA-XY16^40^ of duration 88.9 μs was utilized for the DQ signal excitation and reconversion. A z-filter delay of 1 ms was applied to remove all the residual transverse magnetization before the application of the final 90^o^ read pulse. 32 *t*_1_ and *t*_2_ increments and 24 scans were used. 2D ^1^H/^1^H RFDR spectra were obtained using 64 *t*_1_ increments and 2 scans for a mixing time of 2.84 ms at 90, 80, 70, 60, 50 and 40 kHz MAS. We also implemented the ^1^H/^1^H fp-RFDR pulse sequence during the delay between successive scans to reduce the experimental time as the spin-lattice relaxation times of protons were found to be non-uniform (ranging from 0.9 to 8.0 s) under ultrafast MAS. In practice, ~1.26 times of maximum T_1_ value is usually employed to avoid any signal saturation. However, the recoupling of ^1^H–^1^H dipolar couplings during the repetition delay greatly helps in shortening the repetition delay^42^. In this study, we applied four RFDR trains each with 480 π pulses based on the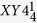 phase cycling scheme. By implementing this method, the repetition delay was reduced to 2 s in the 3D and 2D experiments.

## Additional Information

**How to cite this article**: Zhang, R. *et al.* A Novel High-Resolution and Sensitivity-Enhanced Three-Dimensional Solid-State NMR Experiment Under Ultrafast Magic Angle Spinning Conditions. *Sci. Rep.* **5**, 11810; doi: 10.1038/srep11810 (2015).

## Supplementary Material

Supplementary Information
