# Supplementary material for: A Novel High-Resolution and Sensitivity-Enhanced Three-Dimensional Solid-State NMR Experiment Under Ultrafast Magic Angle Spinning Conditions
Source: Sci Rep. 2015 Jul 3;5:11810. doi: 10.1038/srep11810 (PMC4490345; doi:10.1038/srep11810)
Supplement: Supplementary Information [file srep11810-s1.pdf]

A Novel High-Resolution and Sensitivity-Enhanced Three-Dimensional Solid-State NMR Experiment Under Ultrafast Magic Angle Spinning Conditions

Rongchun Zhang,[1†] Manoj Kumar Pandey,[2†] Yusuke Nishiyama,[2,3]

and Ayyalusamy Ramamoorthy[1*]

[1]Biophysics and Department of Chemistry, University of Michigan, Ann Arbor, MI 48109-1055, USA

[2]CLST NMR Facility, RIKEN, Yokohama, Kanagawa 230-0045, Japan

[3]JEOL RESONANCE Inc., Musashino, Akishima, Tokyo 196-8558, Japan

[†]These authors contribute equally to this work.
*To whom correspondence should be addressed (ramamoor@umich.edu)

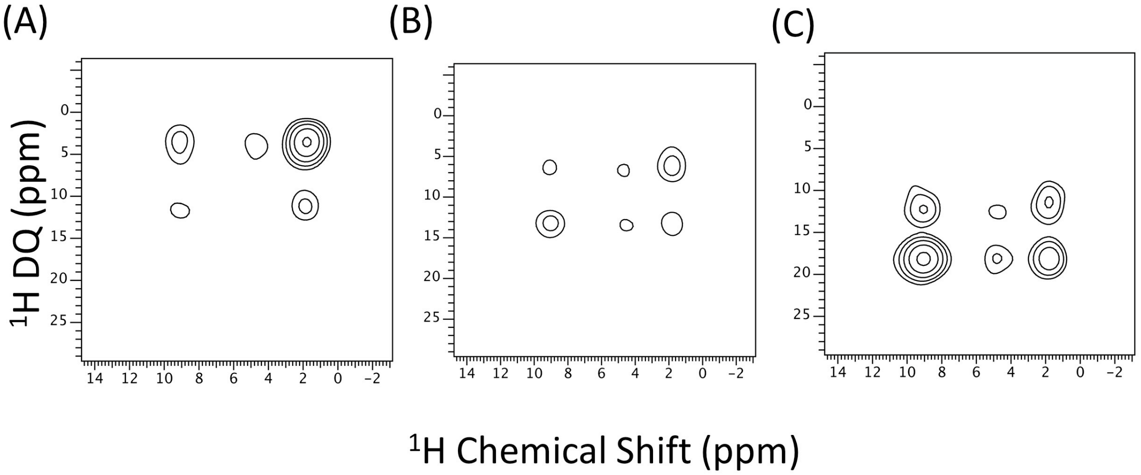

Figure S1. 2D DQ/SQ1 (F2/F1) spectra of a U-^{13}C-^{15}N-L-alanine powder sample sliced along the F3 dimension of the 3D spectrum at proton chemical shift frequiecies of (A) CH_3, (B) CH, and (C) NH_3^+ groups.

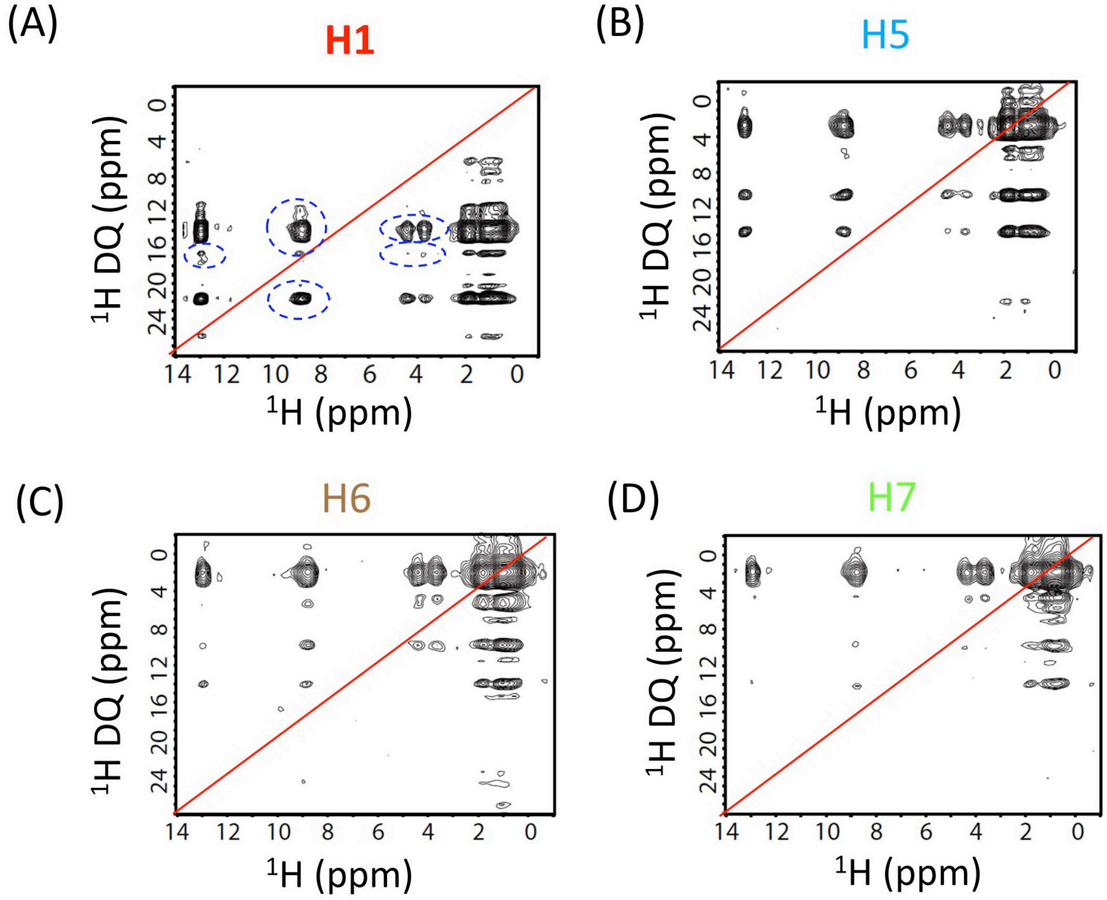

Figure S2. 2D DQ/SQ1 (F2/F1) spectra sliced from the 3D spectrum along the F3 dimension at the proton chemical shift frequencies of (A) H1, (B) H5, (C) H6 and (D) H7. The dashed blue circles indicate the peaks that are absent in the DQ/SQ2 (F2/F3) spectrum.
